# Gut‒heart axis: emerging therapies targeting trimethylamine N-oxide production

**DOI:** 10.1080/19490976.2025.2604868

**Published:** 2025-12-27

**Authors:** Efrain Ricardo Torres, Jennifer Wilcox, W. H. Wilson Tang

**Affiliations:** a Case Western Reserve University School of Medicine, Cleveland, OH, USA; b Department of Heart, Blood, and Kidney Research, Cleveland Clinic Research, Cleveland, OH, USA; c Kaufman Center for Heart Failure Treatment and Recovery, Heart Vascular and Thoracic Institute, Cleveland Clinic, Cleveland, OH, USA; d Cleveland Clinic Lerner College of Medicine of Case Western Reserve University, Cleveland, OH, USA

**Keywords:** Trimethylamine N-oxide, flavin monooxygenase 3, trimethylamine lyase

## Abstract

Trimethylamine N-oxide (TMAO) has garnered considerable attention because of its role in the pathophysiology and pathogenesis of various disorders, particularly heart and kidney disease. Gut microbes produce trimethylamine (TMA) moieties from common dietary precursors, such as choline or carnitine, which are subsequently metabolized by the liver into TMAO. Circulating TMAO then exerts various effects, influencing dyslipidemia, metabolic syndrome, endothelial dysfunction, and inflammation, as well as other detrimental processes. Many existing medications have been shown to decrease TMAO levels in the blood. However, it remains uncertain whether the improved clinical outcomes offered by these medications are related to the reduction in TMAO levels. Additionally, some of the treatment mechanisms do not directly attack the root problem, so other medications have been developed and trialed. Among this latter group of medications, TMA lyase inhibitors have shown a significant ability to decrease serum TMAO without possessing many theoretical downsides. Studies have demonstrated that these medications improve outcomes, including decreased platelet aggregation, improved blood pressure, enhanced glycemic control, a reduced risk of major adverse cardiac events, and a decrease in the rate of renal dysfunction. Because TMAO is involved in a wide range of disease-related biological processes, further research into new therapies is warranted, with potential implications not only for cardiovascular disease but also for cancer or chronic inflammation conditions as well.

## Introduction: TMAO as a therapeutic target

Trimethylamine N-oxide (TMAO) is increasingly recognized as a crucial microbial metabolite linking diet, the gut microbiota, and systemic diseases, particularly those affecting cardiovascular and metabolic health. The structure's central trimethylammonium moiety and oxide group resemble those of many biochemically active molecules, allowing it to form hydrogen bonds and remain water soluble, which contributes to its availability as a metabolite.^1^ Interestingly, it is theorized to help some marine animals maintain homeostasis and protein structure in the presence of intense osmotic pressures and the protein-denaturing nature of urea.^2^ Previously described as a harmless metabolic byproduct, it is now implicated in the pathogenesis of cardiovascular disease (CVD), chronic kidney disease (CKD), and other metabolic syndromes.^3^

To study the extensive connections between TMAO and clinical pathology, genome-wide association studies have been conducted, demonstrating that it is associated with dysfunctions in multiple metabolic, gastrointestinal, immune, arterial, neurological, and cholesterol pathways. Associations based on Mendelian genetics show high scores for various cancers, schizophrenia, and susceptibility to other immune-related disorders.^4^

To gain a deeper understanding of this small molecule and its effects, all pathways involved in its formation should be analyzed. TMAO forms in the liver via the oxidation of trimethylamine (TMA), a product of the gut microbial metabolism of nutrients such as choline, L-carnitine, and phosphatidylcholine, which are abundant in red meat and other animal products.^5^ ^,^ ^6^ The effects of TMAO are multifaceted, often involving inflammatory cascades, and drive atherosclerosis through cholesterol modulation, pro-thrombotic states, and platelet activation, as well as other mechanisms that require further research to elucidate. For one, it disrupts lipid metabolism by reducing reverse cholesterol transport and increasing macrophage foam cell formation through the upregulation of scavenger receptors—key mechanisms in driving atherosclerosis development. Additionally, TMAO activates inflammatory signaling cascades, including the MAPK and NF-κB pathways, thereby promoting endothelial dysfunction and vascular inflammation.^7^ Experiments have also shown that elevated levels of TMAO lead to accelerated platelet activation and an increased likelihood of thromboembolic events.^8^ These findings underscore the relevance of TMAO as both a biomarker and therapeutic target in cardiometabolic diseases.

Improving knowledge of the mechanisms by which TMAO drives atherosclerosis and metabolic syndromes can aid in precise targeting, providing options for future therapeutic interventions. Additionally, existing therapies targeting TMAO formation should be further evaluated to assess their clinical utility and translate these breakthroughs into improved patient outcomes. This paper reviews TMAO, the mechanisms through which it causes disease, and ongoing and prospective therapies to reduce circulating TMAO.

## Microbial and host factors in TMAO synthesis

**Figure 1.**
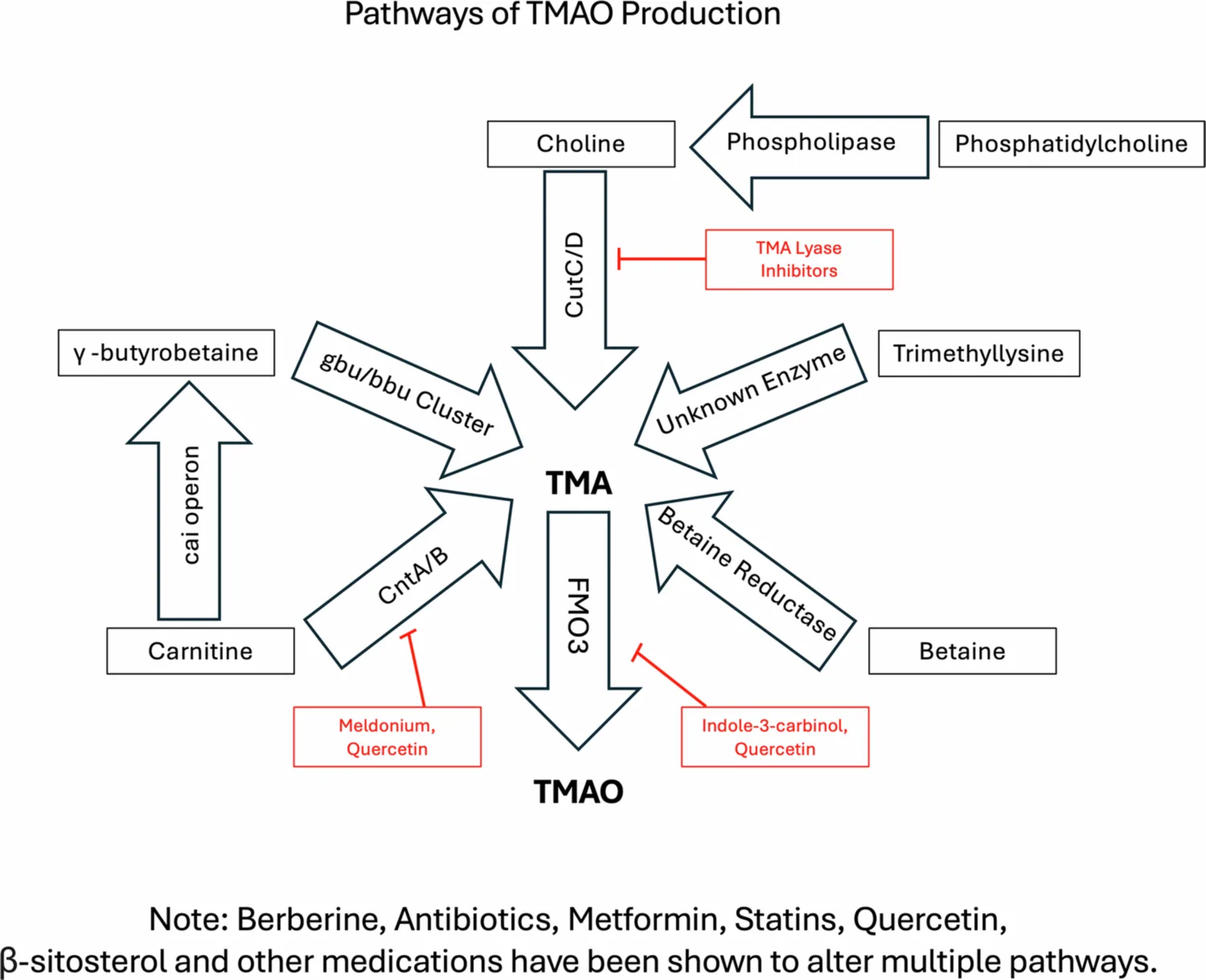
Pathways of TMAO production. Berberine, antibiotics, metformin, statins, quercetin, β-sitosterol and other medications have been shown to alter multiple pathways. Abbreviations: TMAO, trimethylamine N-oxide; TMA, trimethylamine; CutC/D, choline trimethylamine lyase; CntA/B, carnitine monooxygenase; FMO3, flavin-containing monooxygenase 3.

TMAO biosynthesis is a two-step process that begins in the gastrointestinal tract and concludes in the liver. (Figure 1) As such, both microbial and host factors influence TMAO synthesis. Host factors contributing to elevated TMAO include genetic predispositions and dietary intake of foods that contain the relevant precursors. For example, cross-continental studies have shown consistent associations between elevated TMAO and dietary animal protein intake, albeit with some regional variations influenced by cultural dietary norms.^9^ ^,^ ^10^ Bacterial genera, including but not limited to *Clostridium*, *Desulfovibrio*, *Escherichia*, and *Akkermansia*, produce TMA through enzymes such as choline TMA lyases (CutC/D), carnitine monooxygenases (CntA/B), *γ*-butyrate-producing enzymes, and betaine reductases.^11^ Of these, the CutC/D group has been credited with producing the highest proportion of TMAO in mice, originally making it the primary target for therapeutic intervention.^12^ Now, it is understood that the choline sources require more nuance to evaluate; the free forms of choline consistently results in elevated TMAO while the phosphatidylcholine or other bound forms have shown inconsistent results with less TMAO increase.^13-15^ While early studies highlighted the cntA/cntB oxygen-dependent monooxygenase system as a key route for L-carnitine metabolism, more recent evidence indicates that the anaerobic *γ*-butyrobetaine utilization (gbu/bbu) pathway is the dominant contributor under physiological gut conditions. This pathway enables efficient anaerobic conversion of *γ*-butyrobetaine to TMA in the distal gut, where oxygen is scarce, thereby linking dietary L-carnitine and red meat intake more directly to hepatic FMO3-mediated TMAO formation.^16-18^ In contrast, cntA/cntB activity is limited by its oxygen requirement and is unlikely to account for the majority of carnitine-induced TMA production in these regions.^17^ ^,^ ^19^ There is also evidence that trimethyllysine contributes to the total TMAO pool. However, the enzymes involved have not been extensively characterized, unlike those of the other pathways, and their contribution is relatively modest compared to the others.^20^ As the formation of TMA is critical and occurs through intestinal bacteria, it is essential to consider the changes in microbiota that may contribute to elevated TMAO production.

The bacteria involved often thrive in high-protein and high-fat diets, and the substrates for these reactions are derived primarily from dietary animal products. These enzymes increase expression and activity in response to dietary availability, highlighting the essential relationship between the gut microbiome and vascular atherosclerosis. Once TMA is produced, it is rapidly absorbed into the portal circulation and oxidized to TMAO by flavin-containing monooxygenase 3 (FMO3).^5^ The efficiency of this conversion may be modulated by host genetics, particularly by previously described polymorphisms in the FMO3 gene. For example, the E158K genetic variant has been associated with elevated blood TMAO levels and a more rapid decline in kidney function.^21^ Other studies have shown evidence of more than 40 FMO3 variants, some of which can reduce or increase enzyme activity, influencing circulating TMAO levels and disease susceptibility.^22^ Some of these variants have been evaluated for their role in trimethylaminuria, but their role in atherosclerosis driven by TMAO has not been fully assessed. Experiments have also covered oral microbiota-driven effects on FMO3, showing that dysregulation via *P. gingivalis* can elevate serum TMAO levels without changing gut TMAO levels, primarily via increases in hepatic FMO3 activity.^23^ A similar hepatic enzyme, FMO1, is responsible for a portion of TMAO production; however, studies have shown that the amount of TMAO is likely less significant than that of FMO3.^24^ ^,^ ^25^

The resilience, stability, and diversity of the host microbiome also play a role in TMAO homeostasis. Some known gut microbes, such as *Lactobacillus,* have been shown to have endothelial-protective effects, which appear to be opposite to those of TMAO-induced injury.^26-28^ Additionally, examining bacterial ratios within the host microbiome can allow for an estimation of serum TMAO, further highlighting the importance of the host microbiome in TMAO-related disorders.^3^ ^,^ ^29^ This suggests that targeting either microbial prevalence, microbial gene expression or host enzymatic conversion could offer viable therapeutic avenues for lowering TMAO burden. Furthermore, understanding FMO3 genetic variations and their relationship with disease prevalence may provide a deeper insight into the impact of host factors on disease prevalence.

Recent work has expended to support the notion that TMAO can be produced outside the liver, such as in mature adipose cells, which can express FMO3. This expression increases with age, contributing to local and systemic TMAO burden. Additionally, FMO3 expression is associated with white adipose dysfunction, including inflammation and senescence.^30^ These findings are especially significant in aging and patients with metabolic syndrome. After understanding the mechanisms of TMAO synthesis, research can focus on the mechanisms by which it affects health and study these mechanisms in relation to different known disease processes.

## Mechanisms of pathophysiology

TMAO exerts its pathological effects via multiple interrelated mechanisms, including inflammation, endothelial dysfunction, and increased gut permeability, as well as promoting several disease-specific pathways.

### Inflammation

One pathway involved in TMAO-induced inflammation is the activation of protein kinase C (PKC) and NF-kB, increasing the expression of cell adhesion molecules such as VCAM1 and ICAM1, facilitating monocyte recruitment into the vessel wall, and increasing atherogenesis. VCAM1 has also been implicated in the expression of protein arginine methyltransferase 5 (PRMT5), and studies have shown that PRMT5 knockdown ameliorates the vascular inflammation and macrophage adhesion caused by TMAO in vitro.^31^ Elevated TMAO also promotes inflammation by dysregulating the NLRP3 inflammasome through mechanisms such as TLR4 upregulation and increased oxidative stress, paralleling pathways observed in other inflammatory pathologies.^32^ ^,^ ^33^ Finally, TMAO suppresses AMPK, a key cellular energy sensor that normally boosts antioxidant enzymes such as catalase and superoxide dismutase.^34^ ^,^ ^35^ Collectively, it is observed that elevated TMAO results in a multifactor-mediated increase in the expression of adhesion and inflammatory molecules, as well as a decrease in antioxidant capacity, which combine to significantly worsen endothelial function and promote disease. This inflammation is often coupled with endothelial disruption in the pathogenesis of cardiovascular diseases; therefore, both must be considered when discussing the effects of TMAO.^36^

### Endothelial dysfunction and cholesterol handling

Elevated TMAO increases the expression of CD36 and scavenger receptor A on macrophages, which enhances the uptake of oxidized low-density lipoproteins (LDL) and promotes foam cell formation—a hallmark event in atherogenesis.^37^ Additionally, TMAO has been shown to decrease the expression of the ABCA1 transporter, which facilitates reverse cholesterol transport and slows the progression of plaque in murine macrophage cell lines, thereby worsening atherosclerosis development.^38^ TMAO also increases lipid accumulation and reduces cell viability in HepG2 liver cells in vitro and worsens liver damage in high-fat diet mice in vivo, leading to more severe steatohepatitis and lipid accumulation.^39^

Emerging evidence suggests that endothelial cells can generate TMAO themselves, with data revealing extrahepatic expression of FMO3 in human and mouse aortic cells, supporting a local vascular source of TMAO. Locally produced TMAO triggers stress in the endoplasmic reticulum, which spreads to mitochondria and reprograms endothelial cell metabolism toward glycolysis, generating mitochondrial ROS.^40^ In addition, TMAO induces oxidative stress by upregulating nicotinamide adenine dinucleotide phosphate (NADPH) oxidases, specifically NOX2 and NOX4.^41^ NADPH oxidases increase oxidative stress by producing superoxide anions, which can directly damage the endothelium and reduce nitric oxide availability.^42^ ^,^ ^43^ Together, oxidative stress, lipid accumulation, and aberrant cholesterol handling play a crucial role in endothelial dysfunction and atherogenesis, prominent aspects of the negative effects of TMAO in humans.

### Increased gut permeability

Yet, another important part of TMAO pathology is an increase in gut permeability. TMAO has been increasingly linked to metabolic dysfunction-associated steatotic liver disease (MASLD) and leaky gut syndrome because of its association with metabolic disorders. In vivo, mice on a high-fat diet (HFD) supplemented with TMAO exhibited greater colonic mucosal damage, reduced tight junction proteins, and elevated inflammatory markers compared to those on HFD alone, supporting the notion that TMAO induces tissue injury.^39^ This disruption was mediated by HMGB1, a marker of cell damage, and reversed by its inhibition; gut barrier integrity was also restored by silencing TLR4, with which the cell receptor HMGB1 interacts. Increased gut permeability enables harmful substances to enter the circulation and may underlie metabolic disorders, including diabetes, obesity, and cardiovascular disease.^44^ These findings suggest that TMAO-induced gut barrier disruption may play a crucial role in promoting systemic inflammation and metabolic diseases.

### Prothrombotic effects

Some of the cardiovascular-related consequences of elevated TMAO are attributed to its combination of inflammatory and prothrombotic effects. Elevated TMAO enhances procoagulant activity and platelet activation, increasing thrombotic risk, and is, unsurprisingly, a strong predictor of thrombotic events, such as stroke and myocardial infarction (MI). These changes are attributed to changes in stimulus-dependent calcium release from intracellular stores. Exposure to TMAO increases platelet aggregation in both platelet-rich plasma and washed human platelets after stimulation with ADP and thrombin, respectively. Additionally, both the time to thrombosis and the area of adhesion significantly increased after exposure to TMAO in these aggregation experiments.^8^ Other experiments have demonstrated that TMAO-induced procoagulant activity is driven by tissue factor and MAPK pathway activation, which is reversed by blocking either route. Additionally, although it does not cause systemic coagulopathy in mice, TMAO does downregulate thrombomodulin in human endothelial cells.^45^

### Proarrhythmic effect

Further cardiac ramifications imposed by elevated TMAO are proarrhythmic effects. Atrial fibrillation (AF) may also be a downstream effect of elevated TMAO levels and is mediated through the various inflammatory and endothelial-disrupting changes described above. Extensive human cohort studies have shown that TMAO concentration is correlated with the development of AF, even after controlling for dietary intake of choline.^46^ Other prospective studies have shown that elevated TMAO is associated with a higher prevalence of persistent atrial fibrillation compared to paroxysmal atrial fibrillation or sinus rhythm, and that preablation TMAO was independently associated with the recurrence of AF at 1 y.^47^ Additionally, other large cohort studies have shown that high levels of trimethyllysine are associated with an increased risk of incident AF development when patients are followed for more than 10 y.^48^ Taken together, elevated TMAO increases patient risk for developing atrial fibrillation, which becomes even more serious when considering the pro-thrombotic effects of TMAO, which may exacerbate the clinical manifestations of arrhythmias.

### Cancer risk

Given the previously discussed consequences of elevated TMAO, namely, oxidative stress, inflammation, and metabolic disruption, its association with the prevalence and pathogenesis of numerous different cancers is unsurprising. Epigenetic database correlations using Mendelian genetics have identified colorectal cancer as the number one disease associated with elevated TMAO, and it remains in the top 11% of associations when employing a broader pangenomic study method. Other cancers were also significantly associated, with gastric, breast, and ovarian cancers all ranking in the top five most associated diseases based on Mendelian genetics.^4^ High concentrations of TMAO have been reported in samples of oral squamous cell carcinoma,^49^ and serum TMAO levels showed a correlation with hepatocellular carcinoma and aggressive prostate cancers.^50^ ^,^ ^51^ Some of the same mechanisms involved in its link to cancer are prevalent in neurological diseases as well, and this link has been better characterized recently.

### Neurologic effects

TMAO crosses the blood-brain barrier and accumulates in the brain at levels similar to those in serum, unlike its precursors, suggesting it may be a neurologically active compound.^52^ Interestingly, physiological levels of TMAO were found to enhance blood-brain barrier integrity and protect the brain from inflammation but TMA increased brain barrier permeability, implying a complicated relationship between gut and brain health.^53^ In aged mice, antibiotic-induced microbiome suppression lowered both TMAO and oxidative stress, linking gut-derived TMAO to neuroinflammation and neurodegenerative disease.^54^ ^,^ ^55^ TMAO levels naturally increase with age, and providing TMAO supplementation to mice can induce an “aged” phenotype with worse novel object recognition and increased neuroinflammation.^52^ In humans, Alzheimer's patients had elevated CSF TMAO, which accelerates Tau protein aggregation, a key step in Alzheimer's pathology.^52^ ^,^ ^56^ Others have described TMAO's association with other neurodegenerative diseases, showing increases in clusterin and a-synuclein aggregation, hallmarks in Alzheimer's and Parkinson's, respectively.^57^

**Figure 2.**
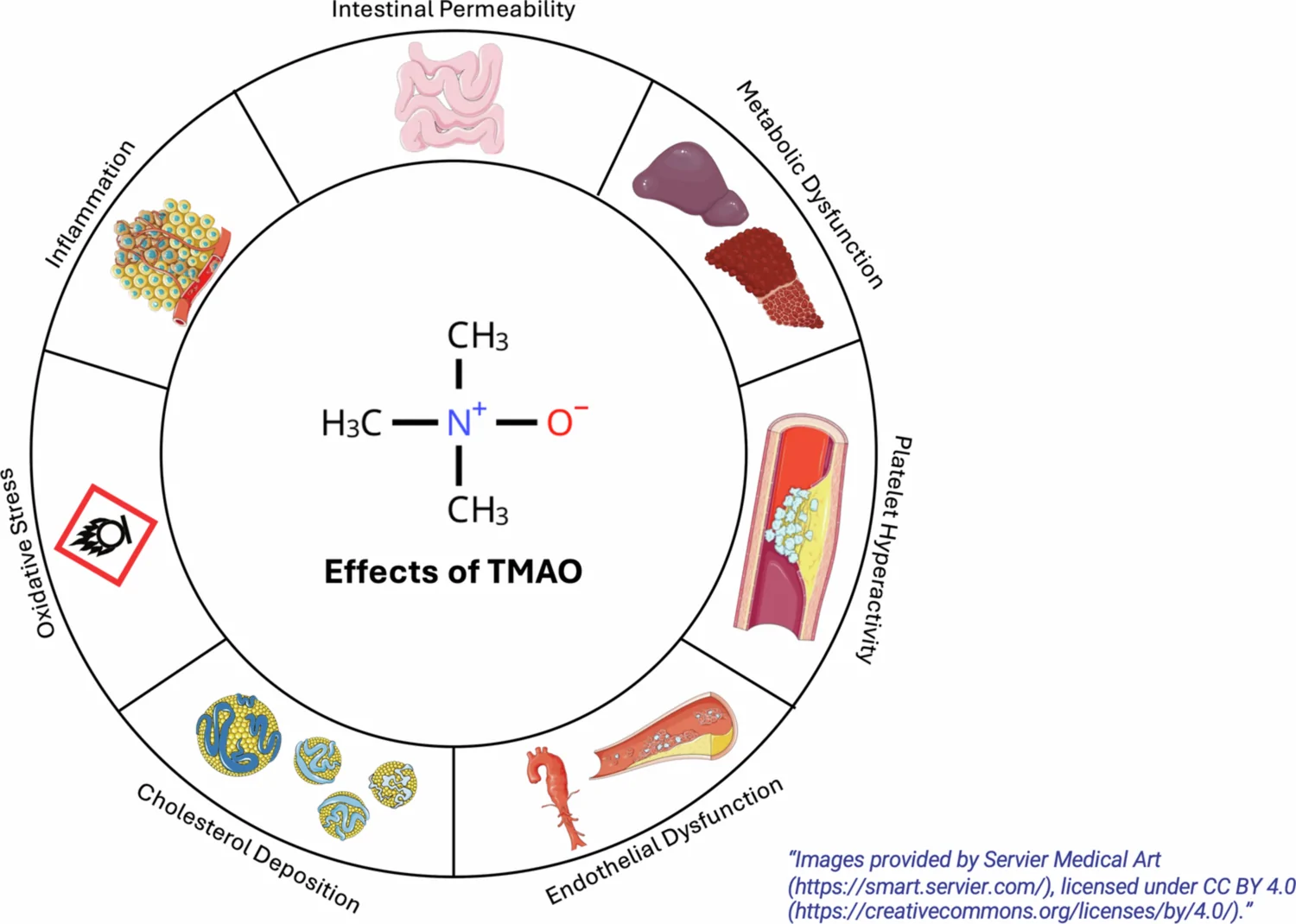
General mechanism of pathophysiology related to TMAO. This illustrates the various TMAO mechanisms that can lead to dysregulation. The small molecule TMAO is shown centrally.

It is acknowledged that many pathological processes (Figure 2) are intricately intertwined with complex connections and interactions to produce a disease state. For example, the combination of inflammation, intestinal permeability, and oxidative stress all likely play a role in the neurologic disorders associated with elevated serum TMAO. Therefore, any treatments targeting elevated TMAO must take this complexity into account.

## Targeting TMAO: dietary changes to eliminate high TMAO precursors

Because TMAO is derived primarily from dietary precursors, specialized diets can be very effective at eliminating it. Many diets, including vegetarian, vegan, keto, and others, have been examined for their effectiveness in improving overall health and reducing TMAO. Studies have investigated the relationship between various foods and TMAO, with saturated fats and animal proteins, especially fish, red meat, and eggs, showing a positive correlation. In contrast, the intake of nuts and plant protein were both negatively correlated with TMAO levels.^9^ In the case of eggs, which contain high levels of the precursor molecule choline, one interventional study found that the source of choline had a significant effect on TMAO levels and downstream effects, as platelet hyperactivity and TMAO levels were elevated only in participants that took a choline bitartrate supplement but not in those who consumed the equivalent amount of choline in whole eggs.^15^

### Red meat intake

The consumption of red meat has frequently been linked to an increase in the serum TMAO, likely due to its abundance of known TMAO precursors. However, some experiments have not included adequate time intervals to allow for endogenous carnitine-derived TMAO production from red meat intake. One article reported that seafood intake was significantly correlated with elevated TMAO, whereas red meat intake was not; however, seafood contains preformed TMAO, resulting in a prompt postprandial increase, whereas red meat requires a multistep synthesis and increases concentrations around 12–24 h.^29^ ^,^ ^58^ The same study revealed that an unhealthy diet, characterized by higher intake of less-healthy plant based foods like fruit juices, sugar-sweetened beverages, refined grains, and desserts^59^ was inversely associated with TMAO levels, indicating that it is not simply a marker of a healthy or longevity-sustaining diet, but instead has a more complex relationship with health and nutrition.^60^ Another extensive analysis took it a step further and examined different papers reporting on TMAO and diet, which included studies showing TMAO increased with red meat intake, as well as some that found no significant difference.^61^ Many of those experiments that had trouble finding an increase in TMAO after carnitine challenges lacked adequate timing or used only a moderate amount of red meat, whereas studies that included a significant carnitine load from red meat have shown clearer TMAO elevation.^62^ A trial on the Mediterranean diet showed that reduced red meat intake was more effective in reducing TMAO than the same diet with increased red meat intake.^63^ Overall, there is a substantial distinction in protein sources and their potential relationship to TMAO levels: reducing red meat intake lowers carnitine-induced TMAO, and red meat intake exhibits different kinetics compared to seafood intake due to the difference between preformed and endogenously formed TMAO.

### Vegan and vegetarian

Other popular diets aimed at improving overall health include the vegan and vegetarian diets, which have been studied in the context of TMAO, as some of its precursors are significantly decreased in these diets. An 8-week vegan diet successfully decreased plasma TMAO levels, but these levels rebounded after a 4-week resumption of the regular diet. In contrast, postprandial glucose remained significantly reduced even after the resumption of the regular diet for 4 weeks.^64^ An observational study found that the levels of TMAO and TMA were not significantly different between groups of lacto-ovo-vegetarians and vegans.^65^ This finding may challenge the earlier notion that animal-derived choline is the largest source for TMAO production in the human gut. Other studies have shown that vegans and vegetarians have a higher adherence to the Mediterranean diet, which is associated with decreased serum TMAO.^66^

### Mediterranean diet

Decreasing red meat consumption in a Mediterranean diet more effectively reduces TMAO, and vegans and vegetarians tend to have better adherence to these diets. Adherence to the Mediterranean diet is positively correlated with a high-fiber diet index, influences the gut microbiota composition without altering diversity, and increases the production of short-chain fatty acids. Metagenomic analyses also revealed enriched metabolic pathways in these subjects related to carbohydrate and SCFA metabolism when compared to those with less adherence to the Mediterranean diet. These effects on the gut, as well as the types of food found in the Mediterranean diet, help explain why lower TMAO levels have been observed in patients following the Mediterranean diet.^66^

However, one randomized controlled trial showed that following a Mediterranean diet did not reduce serum TMAO in patients at risk for colon cancer.^67^ These mixed results suggest that certain aspects of this diet likely play a greater role in reducing TMAO than others. For example, the Mediterranean diet is traditionally low in red meat, high in seafood, and high in extra virgin olive oil, which contains an inhibitor of TMAO production. Further complicating the matter, data suggest that the type of fish or meat consumed is important, with saltwater fish, dark meat, and shellfish being associated with increased TMAO levels, whereas freshwater fish do not have the same consequences.^68^ These nuances must be taken into account when validating dietary interventions or recommending changes to treat elevated TMAO. In addition to the sources and composition of our diet, overall caloric consumption plays a role in the amount of precursors available to the gut microbiota involved in TMAO production.

### Caloric restriction

As many metabolic problems arise from a general caloric surplus, caloric restriction has been a mainstay in dietary intervention since the inception of dieting. Choline is found in all foods, so the amount of food consumed can contribute to TMAO levels as much as the makeup of the diet. One study showed that a brief caloric reduction was more effective at reducing TMAO levels than adding vegetables to a normal diet.^69^ Low-calorie diets have also been tested, revealing that a hypocaloric group showed a significant average decrease in TMAO when compared to a group with normal caloric intake, which showed an increase.^70^ From these results, it is reasonable to conclude that caloric surplus should still be avoided in at-risk patients who seek to reduce their TMAO levels to improve health.

### Refined foods

Another important dietary factor studied for its role in TMAO levels is the consumption of refined foods. Interestingly, in one analysis, the levels of TMAO were significantly higher in diets containing whole-grain cereals compared to refined cereals and also considerably higher in diets containing high amounts of biologically active omega-3 fatty acids.^71^ Another study furthering the complex relationship between diet and TMAO revealed that a high-fiber resistant starch diet has been shown to increase plasma TMAO levels, yet these elevations were not associated with markers of atherosclerosis, such as coronary artery calcium or carotid intima-media thickness.^72^ This highlights that elevated TMAO may not uniformly translate into increased cardiovascular risk across all contexts, and the other health benefits of consuming refined foods or “healthy” fats should be considered in the context of a patient's normal diet rather than as a standalone risk marker.

An additional dietary element with a proven association with TMAO production is high salt intake; however, the amount of TMA remains unchanged even as TMAO increases,^71^ highlighting the importance of understanding the pathophysiology before addressing this problem.^73^ Experiments demonstrated that dose-dependent increases in sodium intake led to increased plasma TMAO, accompanied by decreased kidney TMAO excretion. Additionally, the gut microbiota composition after high-salt intake showed significant changes in 21 different genera, as well as dose-dependent changes in specific genera of interest, such as Lactobacilli.^73^ The decrease in Lactobacilli could be particularly interesting, as other experiments have hailed it as a protective factor in cholesterol-induced vascular injury, among other disease states, as discussed above.^28^ ^,^ ^74^ In rats, high salt intake leads to increased blood pressure and TMAO levels, which are partially alleviated by treatment with the TMA lyase inhibitor DMB.^41^

### Food timing

One last important consideration when discussing TMAO and dietary effects on health is the “French Paradox.” Essentially, there are geographic regions with a drastically “unhealthier” diet than their longevity and health statistics would suggest, such as those of the European countries France and Italy. Their traditional diets contain high levels of saturated fats, yet their prevalence of cardiovascular disease does not reflect that. There are many theories regarding the various foods consumed in these diets and how they may influence health, such as the idea that moderate wine consumption could improve stress and overall health. Possibly, the most common current hypothesis is that their health outcomes are the result of numerous variables, from daily walking to the reduced speed at which they eat. Studies on eating speed have found that eating slowly provides more meal enjoyment and leads to less snacking in the period after eating.^75^ These effects likely have a significant hormonal basis, as other studies have shown an increase in appetite-regulating hormones, such as peptide YY and GLP-1.^76^ While this research provides strong evidence for the health benefits of slower eating, to our knowledge, there are no published data on the relationship between eating pace and TMAO levels. Given the strong relationships among TMAO, eating habits, and cardiovascular health, it would be interesting to explore this further and evaluate the levels of TMAO in various timed eating regimens.

## Targeting TMAO: common cardiometabolic drugs

After considering the important dietary measures taken to reduce TMAO, some existing medications may offer benefits. Notably, some cardiometabolic medications already used in common TMAO-related comorbidities have been proven to decrease TMAO through various mechanisms.

### Aspirin

Currently, clinical guidelines from the American Heart Association and American College of Cardiology do not recommend the use of aspirin as an antiplatelet therapy except in patients who have preexisting coronary artery disease.^77^ The same guideline states that the current literature shows a balance between the bleeding risk of aspirin and its cardiometabolic benefits but that a large primary prevention trial is already underway. However, low-dose aspirin can partially reduce TMAO and its associated prothrombotic effects, particularly those induced by dietary choline.^78^ Aspirin has already been shown to influence the gut microbiota composition, and the microbiome effects on TMAO production have been thoroughly described.^79^ ^,^ ^80^

### Glucose-lowering medications (Metformin, SGLT2i)

Other cardiometabolic medications that affect TMAO include some glucose-lowering medications, such as metformin and SGLT2 inhibitors. Metformin has been shown an ability to decrease TMAO levels in diabetic mice on both a chow diet and a choline-supplemented diet. Metformin treatment was also able to reduce the production of TMA by the bacterial species *Klebsiella pneumoniae* and *Proteus mirabilis,* especially *P. mirabilis*. The expression of the genes that encode TMA lyase was not statistically significant after the administration of metformin; however, signaling that metformin may inhibit the production of TMA through a different mechanism or that it may be multifactorial.^81^ Sodium glucose cotransporter 2 (SGLT2) inhibitors have also been evaluated for their effects on TMAO, revealing surprising results. Despite decreasing cardiac remodeling, major adverse cardiac events, and insulin resistance, data suggest that they increase serum TMAO through unclear mechanisms.^82^

### Statins

With increasing evidence illustrating the connection between TMAO and common cardiovascular and cardiorenal disorders, several heart-healthy medications have been evaluated for treatment possibilities. Statins, for example, have been associated with a decrease in TMAO in multiple studies, including those from cardiac catheterization registries.^83^ ^,^ ^84^ Some studies have conducted in-depth examinations of the underlying mechanisms, testing statin medications on the abundance of TMAO and its precursors. One demonstrated that levels of TMAO decreased with rosuvastatin administration, while the concentrations of the precursors increased. This raises questions about the mechanisms by which statins can decrease TMAO, specifically whether there is a change in TMA lyase activity.^85^ Another study closely examined the changes in the gut microbiota and genetic expression and reported that the microbial richness and diversity did not change by a statistically significant amount. The expression of genes responsible for producing TMAO decreased, while the concentrations of multiple TMA precursors increased to varying extents. Another interesting finding was that patients who experienced a worse improvement in cholesterol levels also had a significantly increased serum TMAO.^86^ Delving into the existing cardiometabolic medications, angiotensin-converting enzyme inhibitors and angiotensin receptor blockers have also been evaluated for their role in TMAO production and pathophysiology.

### Angiotensin-converting enzyme (ACE) inhibitors/angiotensin receptor blockers (ARBs)

A first look at the mechanisms of both ACE inhibitors (ACEIs) and ARBs may lead you to think that they will worsen TMAO levels and related diseases. TMAO is almost exclusively excreted through glomerular filtration, and meta-analyses have established correlations between decreased renal function and elevated TMAO levels.^87^ Because ACEIs and ARBs can dilate the efferent arterioles and decrease glomerular filtration pressure, one could hypothesize that their administration would result in an increase in serum TMAO levels and subsequent disease progression via the previously discussed mechanisms. Fortunately, their nephroprotective effects can delay kidney injury, decrease the progression of fibrosis, improve solute handling in the kidney, and reduce TMAO-induced inflammatory damage. Additionally, ACEIs and ARBs directly counteract the toxic uremic effects of TMAO, which normally downregulates the expression of megalin, a crucial protein in renal albumin handling.^88^ This rescues the albumin loss experienced in high-TMAO states, protecting the kidney and preserving protein. While the interaction is complex, studies have shown that ACEIs and ARBs can decrease serum TMAO levels and increase urinary excretion of TMAO.^89^ Even after all the research into cardiometabolic medications, there remains a new class of medications, novel targets specifically designed for TMAO.

## Targeting TMAO: novel targets

As TMAO is a small molecule associated with or involved in the pathogenesis of numerous diseases, it is increasingly recognized as a hot target for therapy, especially in the field of preventive medicine. Its biochemical pathways and relevant enzymes have recently been studied more, allowing us to gain a better understanding of the possible ways to decrease its production or increase its excretion, thereby modulating long-term health from multiple standpoints and body systems. Notably, there are a couple of main points in the TMAO production pathway that can be targeted to decrease its availability. One is TMA lyase, the enzyme responsible for converting choline into TMA. This pathway is responsible for most TMA formation in mice, so it may greatly reduce TMAO.^90^ As this step is upstream of TMA production, it allows us to decrease TMAO without increasing the levels of TMA, which is known for its fishy odor in some genetic disorders.^91^ By inhibiting the TMA lyases Cut C and Cut D and thereby inhibiting TMA production, these compounds reduce circulating TMAO levels, mitigating its adverse effects on cardiovascular and metabolic health. As the enzymatic composition of the gut microbiome has become better understood, it has enabled more targeted approaches and improved the prospects of drug therapies. Currently, this is one of the most attractive methods for addressing treatment of elevated TMAO, and extensive research has been conducted in this area. Significantly, these compounds can effectively mitigate the effects of elevated TMAO without incurring a high risk of bacterial resistance to treatment, thereby further increasing their potential.^92^

Similarly, CutC/D inhibitors are compounds that can inhibit CntA/B monooxygenase, thereby decreasing the amount of TMAO created from the precursor carnitine rather than choline. However, in humans, it is likely that the anaerobic gbu/bbu pathway is responsible for more carnitine-induced TMAO production, so this target should be evaluated carefully.^17^ Betaine reductases have also been implicated as potential targets; however, some studies have shown that the amount of TMAO produced from betaine is significantly less than that from carnitine or choline.^12^ Based on the currently available information, TMA lyase inhibitors could be the most promising candidates.

Another strategy for decreasing TMAO is increasing its excretion, primarily through enhanced glomerular filtration or extracorporeal membrane filtration. ACEIs or ARBs can improve glomerular filtration by reducing long-term inflammatory damage to the renal tubules, as described above. TMAO can be removed through renal excretion by modulating transporters, such as OCTN2 and organic anion/cation channels; however, certain medications (e.g., furosemide) may paradoxically increase levels.^93^ ^,^ ^94^ Additionally, extracorporeal filtration, such as dialysis, effectively clears TMAO, suggesting potential clinical benefits in acute settings, despite the current lack of formal guidelines.^95^ A more extreme treatment option is kidney transplantation, which does not reduce TMAO production but does increase its clearance. TMAO levels decrease significantly after kidney transplantation but remain above normal, and elevated TMAO post-transplant is linked to increased mortality and may improve prediction models for graft rejection compared to traditional methods.^96-98^

Other potential treatment mechanisms involve disruption of the gut microbiome, which contributes to the creation of TMAO. This includes medications such as antibiotics, statins, metformin, or any other class that can alter the concentration of the different genera involved. The last step in the production of TMAO can also be targeted, albeit with the known side effects of elevated TMA levels. The molecule TMA is associated with a “noxious” fish-like smell, and genetic abnormalities that decrease basal levels of FMO3, thereby increasing TMA, cause this same symptom. ^22^ Because of this, treating elevated TMAO with FMO3 inhibitors is not an appealing option.

## TMA lyase (Cut C/D) inhibitors

As discussed, TMA lyase is often considered one of the most promising targets, likely because it is responsible for the largest portion of TMAO production in mice.^90^ Screening for inhibitors targeting the TMA lyase enzymes was conducted by introducing the code for the *P. mirabilis* CutC/D enzymes into *E. coli* cells and used for enzyme production. Many substrate lookalikes were screened, and finally, two choline analogs proved to be effective inhibitors of these enzymes. Of the two candidates, 3,3-dimethyl-1-butanol (DMB) was thought to possess a less toxic structure, so it was advanced for further screening and testing in diet-induced TMAO studies.^92^

Different diets, either representing a “Western diet” or one supplemented with additional choline and carnitine, were used to increase the serum TMAO in mice and induce the known health consequences discussed previously. In these mice, DMB reduced their TMAO levels, leading to decreased macrophage foam cell formation and atherosclerotic lesion development without altering cholesterol levels.^92^ ^,^ ^99^ Thus, using compounds such as DMB to block microbial TMA formation can lower TMAO levels in the blood and reverse its harmful effects, based on findings from both animal and human studies.^92^

DMB also attenuates intimal hyperplasia and vascular dysfunction in a mouse model of carotid artery ligation by reducing inflammation and oxidative stress.^100^ Thrombosis and platelet aggregation studies were conducted to evaluate the efficacy of various TMA lyase inhibitors in rescuing prothrombotic states induced by TMAO. The time to occlusion and maximum aggregation were both significantly elevated with the administration of choline, which improved with DMB, and these effects lasted up to 3 d after a single dose of DMB. In mouse models of hind limb ischemia, TMAO worsened perfusion recovery, capillary density and increased oxidative stress and inflammation. DMB administration in these models decreased TMAO and alleviated these effects, increasing angiogenesis, VEGF, and cGMP.^101^

Regarding platelet activity, recent mouse studies have shown that TMAO or choline-induced TMAO increases CES1 expression, increasing clopidogrel hydrolysis and reducing the formation of its active form, limiting its antiplatelet effects. These effects are attenuated by DMB, reversing CES1 induction and restoring active antiplatelet medication activity.^102^ This finding has significant clinical consequences, as many patients taking clopidogrel rely on it to reduce platelet activity for coronary and vascular prophylaxis.

Researchers have sought to learn more about the benefits of TMAO inhibition in other related fields, such as heart failure. To test the potential of DMB in attenuating heart failure remodeling, “overload” mouse models were created through aortic banding surgery and treated with DMB in their drinking water. This treatment reduced the injury-induced increases in hypertrophy, fibrosis, inflammatory markers, and electrical remodeling.^103^ Another disease linked to elevated TMAO is preeclampsia. Studies have shown that fecal microbiota transplantation from a preeclamptic patient to an antibiotic-treated mouse developed similar symptoms related to oxidative damage and that these changes were alleviated by DMB.^104^ These targets are not part of the original hypotheses surrounding endothelial and renal dysfunction, but it is exciting to see how many new disorders can be managed through the modulation of TMAO. Going forward, it will be interesting to see how many different diseases can be improved by TMA lyase inhibitors.

DMB has also been tested for its efficacy in diseases with a less direct link to TMAO, such as neurodegenerative diseases. Mice prone to neurodegeneration were treated with DMB, which increased memory, decreased amyloid aggregation, and decreased inflammation. It also reduced the protein expression of b-secretase, a key enzyme in driving amyloid aggregation and neurodegeneration.^105^ This study demonstrated that inhibiting the TMAO production pathway could yield positive results and improve neurogenerative diseases, a problem that has long plagued the medical field. Another interesting application was demonstrated in mouse studies on collagen-induced arthritis, which also evaluated DMB, finding that it significantly improved arthritis but did so independently of the gut microbiome. Surprisingly, DMB was found to be metabolized into another form, with butyric acid in its structure, and this molecule was also enough to improve arthritis when given separately from DMB. The authors stated that there was no significant difference in cecum TMA with DMB administration, but another TMA lyase inhibitor, fluoromethylcholine (FMC), did inhibit TMA lyase. In another surprising finding, DMB significantly improved the arthritis, while FMC worsened the disease.^106^ Given these findings, further research is required before these medications can be implemented for indications such as collagen-induced arthritis.

Other members of the TMA lyase inhibitor family include iodomethylcholine (IMC) and the previously mentioned FMC, both of which were discovered in efforts to develop a nonlethal inhibitor of CutC/D. As expected, they offer many of the same benefits as DMB, but with different structures, they provide different efficacies for the many enzyme variants harbored by the gut microbiome. Experiments with different TMA lyase inhibitors indicated that their inhibition of CutC/D varies across microbes, with IMC showing more potent inhibition than DMB in both *P. mirabilis* and *D. alaskensis*.^19^ Regardless, both structures were advanced to evaluate their effects on reducing TMAO and mitigating its negative effects, as described above.

Research began on cholesterol-driven changes and revealed that IMC treatment increases fecal neutral sterol loss and reduces intestinal sterol transporter NPC1L1 expression, thereby preventing diet-driven hepatic cholesterol accumulation and altering bile acid metabolism.^107^ IMC also improves renal function and reduces tubulointerstitial fibrosis in a murine model of chronic kidney disease by blocking choline diet-induced elevation in TMAO.^108^ Studies in arrhythmia-prone transgenic mice have demonstrated that the faster onset of atrial fibrillation (AF) seen with choline supplementation compared to control can be ameliorated by the introduction of IMC and even improved the choline-supplemented mice's AF-free survival beyond the survival of control mice who do not receive choline.^109^

Like other TMA lyase inhibitors, researchers have sought to evaluate the effects of FMC on arterial dysfunction. In mouse models of arterial injury, FMC improves TMAO-enhanced thrombogenicity by inhibiting the expression of tissue factor in vascular endothelial cells.^110^ FMC also attenuates abdominal aortic aneurysm formation by reducing endoplasmic reticulum stress-related pathways in the aortic wall.^111^ Combined, these synthetic TMA lyase inhibitors have shown promise in their ability to reduce TMAO and improve the pathogenesis associated with it.

Others have begun to look beyond the synthetics, at some molecules that naturally occur in many plants or even some wines. Berberine, a naturally occurring plant compound used in traditional Chinese medicine, has been found to decrease the levels of TMAO in hypertensive patients at one and three months, with evidence that it does so by inhibiting TMA lyase. The results of mouse studies also reflected this finding, with dose-dependent decreases in both TMAO and blood pressure after berberine exposure. Histological analyses revealed a reduction in aorta thickness in both the berberine and DMB groups compared to the controls. Berberine was also effective at decreasing the concentration of several culprit microbes that express TMA lyase, such as Firmicutes, in a dose-dependent manner.^112^

Another known polyphenolic compound, resveratrol, increases the expression of FMO3 and decreases intestinal production of TMA after choline supplementation by about 60%, likely via TMA lyase inhibition. Resveratrol also attenuates TMAO-induced atherosclerosis and cholesterol dysregulation.^113^ A recent study evaluated a natural polyphenol-rich supplement containing resveratrol and quercetin, which significantly improved maximal walking distances in patients with peripheral artery disease and markedly reduced plasma TMAO levels.^114^ The same supplement was further tested in combination with L-arginine, which improved flow-mediated vasodilation, synergistically enhanced nitric oxide activity and vasorelaxation, and lowered arterial blood pressure in hypertensive models.^115^ These findings may suggest protective effects on endothelial function from these nutraceuticals, and further research is needed to investigate their benefits in humans.

Similar to the polyphenolics, many flavonoids have captured the interest of researchers for their TMA lyase inhibition. One paper employed virtual screening to identify numerous flavonoids that could potentially inhibit TMA lyase and subsequently identified those with promising molecular docking results. These molecules were each discussed for their existing research in cardiovascular benefits, showing that there are likely dozens, if not more, researched structures that could inhibit TMA lyase as part of their mechanism for improving health.^116^ Another paper employed similar methods, followed by assays to confirm that their inhibitor targeted TMA lyase.^117^

Overall, the TMA lyase inhibitors appear to possess considerable potential and should be further evaluated, particularly as some studies have demonstrated health benefits that are independent of the gut microbiome.

### Carnitine monooxygenase (CntA/B) inhibitor

As discussed above, an additional important mechanism that can be modulated is carnitine monooxygenase, which is responsible for producing TMAO from dietary carnitine in aerobic conditions. Meldonium is classified as a CntA monooxygenase inhibitor and prevents carnitine metabolism into TMA, potentially making it effective in decreasing TMAO; however, with mixed results in animal studies have been reported thus far. A study on meldonium administration alongside carnitine administration showed that the carnitine concentration decreased over time, with a compensatory increase in gamma-butyrobetaine. This was explained as an inhibition of carnitine use, resulting in the shuttling of carnitine to the other side of the pathway as gamma-butyrobetaine. Ultimately, one experiment with pretreatment of carnitine for 2 weeks followed by subsequent treatment with labeled carnitine and meldonium revealed significantly decreased plasma TMAO at time points 1, 2, and 4 h.^118^

Similar to TMA lyase inhibitors, researchers have sought out many phenolic and natural compounds that may reduce TMAO levels. Of these, they identified feruloylputrescine, a compound in the orange peel polar fraction, which inhibits TMA production by suppressing the CntA/B enzyme. This has shown potential in alleviating cardiovascular diseases by modulating CntA/B and FMO3 enzymes without directly influencing the gut microbiota composition.^119^

Another is Quercetin, a flavonoid compound that inhibits both FMO3 and CntA/B. Zhang et al. found that it effectively inhibited the adverse effects of a high-carnitine diet by inhibiting CntA/B, resulting in approximately 50% less TMAO at the highest dose tested.^120^ Over several studies, quercetin has been shown to improve lipid metabolism, decrease inflammatory markers, lower liver enzyme markers (AST/ALT), reduce atherosclerotic lesion and plaque size, and decrease the abundance of “harmful” microbiota, such as Firmicutes.^120^ ^,^ ^121^ Many of these effects align with the previously described benefits of removing TMAO, suggesting that it could provide an effective means of doing so. Although the CntA/B enzymes are responsible for a smaller portion of intestinal TMAO production than CutC/D and likely have a more active counterpart anaerobic pathway through gbu/bbu, they continue to be researched for their potential to improve health.^17^ ^,^ ^90^

### FMO3 inhibition

An additional enzyme involved in TMAO production is FMO3, which catalyzes the final step in TMAO production, so its inhibition initially appears to be a sensible target. However, as discussed earlier, the direct inhibition of FMO3 increases TMA levels, which are associated with an unpleasant “fish” odor, thereby making them undesirable treatment options despite having some promise for lowering TMAO. In mice, FMO3 knockout successfully increased TMA, decreased TMAO, and reduced platelet responsiveness and clot formation.^122^ While methimazole has been reported as an inhibitor of FMO3, its clinical use is primarily as an antithyroid medication, and it has severe hepatotoxic side effects, eliminating it from reasonable contention in the treatment of elevated TMAO.^123^ ^,^ ^124^ Alternative FMO3 inhibitors include several different chemical breakdown products of the vegetable compound indole-3-carbinol (I3C). In one trial, patients were provided with I3C by consuming 300 g of Brussels sprouts daily; their TMA-to-TMAO ratios increased by approximately 250%, correlating with a decrease in TMAO of 15%–20%.^125^ Although this may offer potential health benefits, the risks of FMO3 inhibition and subsequent TMA accumulation must be considered as well.

### Fecal microbiota transplantation

As the entire concept of TMAO production and the gut–heart–kidney axes revolve around the intestinal microbiome, transplant studies have piqued the interest of many. Importantly, feces from humans with high TMAO levels transferred to germ-free mice on a choline-deficient diet yielded elevated serum TMAO and the expected subsequent proatherogenic effects, demonstrating that fecal transplantation can affect systemic TMAO levels.^126^ However, fecal microbiota transplantation from lean vegans to patients with metabolic syndrome did not yield a significant change in gut TMAO production and failed to improve the signs of endothelial dysfunction.^127^ This does not mean that fecal transplantation is incapable of improving patient outcomes; however, as many medication regimens have already shown promise and improvement in animal models, they are more promising research targets at this time.

### Fiber, polyphenol, and sterol consumption

Recognizing that TMAO production is a complex issue rooted in the gut microbiome, efforts have been made to either reduce TMAO consumption or promote a beneficial environment to alter the overall microbial composition. One double-blinded, randomized trial investigated the effects of probiotic supplementation in young males, finding that 4 weeks of supplementation did not result in a significant change in TMAO after a phosphatidylcholine challenge. However, the probiotic group did have a greater number of participants who showed a decrease in TMAO after the intervention.^128^ In another study, probiotic strains known for their lipid-lowering effects were tested to evaluate the changes in the concentration of TMA and TMAO. Five of the tested strains showed a decrease in the basal level of TMA in a medium where the only carbon source was TMA. Several of these strains also showed a decrease in mice cecum TMA levels, as well as a significant reduction in choline-induced cecum and serum TMA levels. Among these strains, subspecies of *Bifidobacterium animalis* and *Lactobacillus rhamnosus* also demonstrated decreased hepatic lipid accumulation and restored the homeostasis of cholesterol-regulating genes, which were disrupted by a high-choline diet.^129^ The consumption of polyphenols, which are known for their antioxidant and anti-inflammatory properties, has been shown to increase the abundance of both of these strains and decrease the amount of harmful bacteria, such as Clostridia.^130^ In another preclinical study in mice, supplementation with freeze-dried blueberries decreased serum TMAO levels without altering choline or TMA levels, while increasing gut bacteria negatively associated with TMAO. In contrast, strawberries showed no change in TMAO, which they attributed to differences in the chlorogenic acid and phenolic contents between the two fruits.^131^ Pomegranate extracts have also been shown to decrease choline and carnitine-induced TMA production in human fecal samples.^132^ Taken together, these studies add to the existing evidence that phenolic compounds could help reduce overall TMAO levels by modifying the gut microbiome. Other polyphenols discussed above have been identified as strong inhibitors of enzymes in the TMAO synthesis pathway and are still being researched for their health benefits.

To assess the impact of polyphenols and fatty acids on cardiac biomarkers and metabolic risk, the ETHERPATHS study investigated changes in the serum TMAO following dietary interventions that include combinations of high and low polyphenol and high and low levels of omega-3 fatty acids. The high omega-3 fatty acid and low polyphenol groups had the highest serum TMAO levels, while the low omega-3 fatty acid and high polyphenol groups had the lowest serum TMAO; however, there was no statistical significance regarding the polyphenol dietary intervention.^71^ While modulating the gut microbiome through prebiotics, probiotics, and polyphenols is an interesting approach to reduce TMAO production, the consistency and clinical significance of these effects remain uncertain, highlighting the need for further targeted and mechanistic studies.

Garlic organosulfur compounds, such as allicin, have been shown to be potent antimicrobial agents, including the action against the TMA-producing bacteria mentioned above.^133-136^ These compounds can decrease carnitine-induced increases in TMAO and plaque burden, resulting in an increase in butyrobetaine, which suggests that they may inhibit the aforementioned anaerobic gbu/bbu cluster enzymes.^135^ ^,^ ^136^

**Figure 3.**
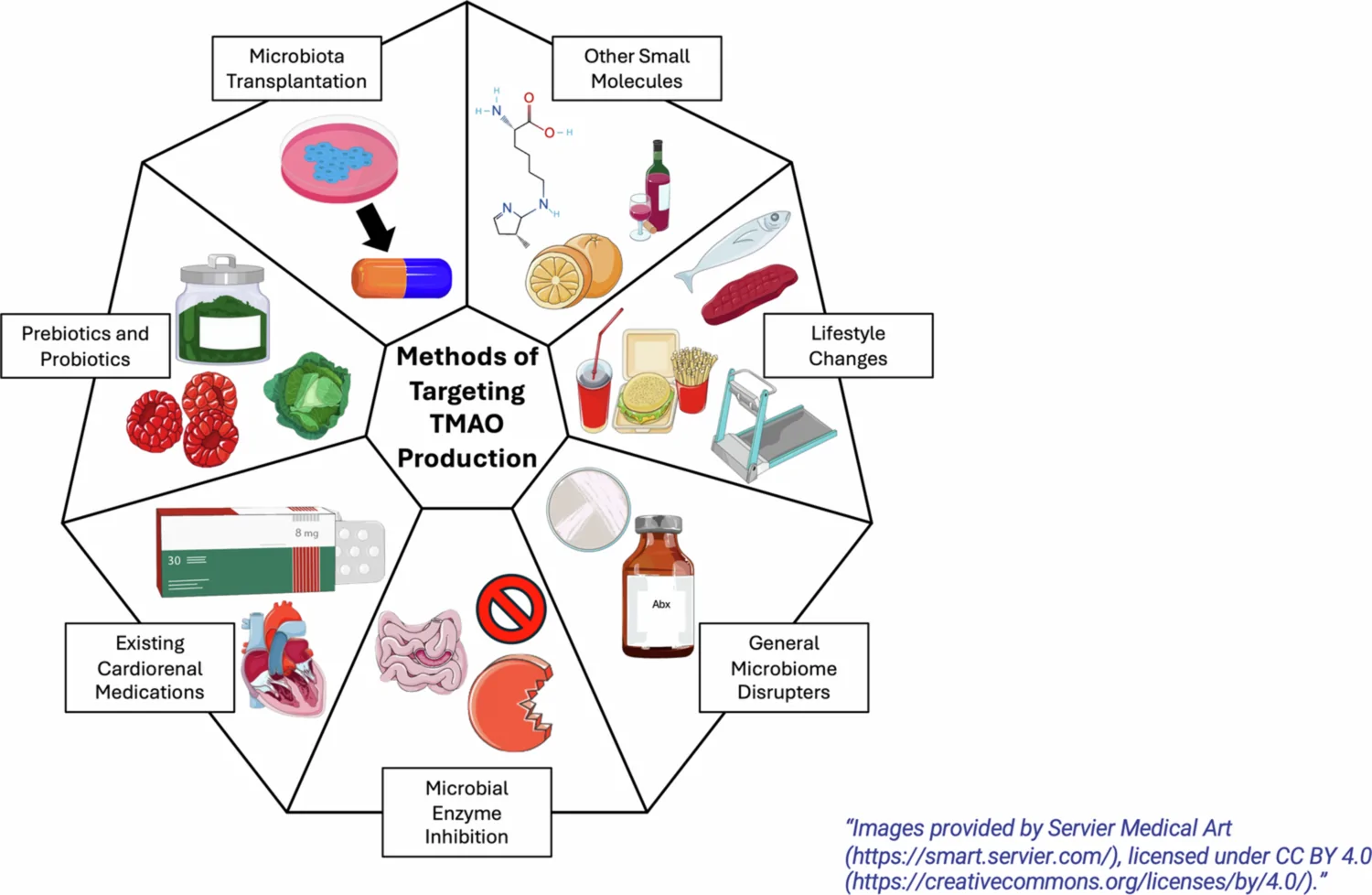
Different methods of targeting TMAO production. This illustrates the various methods that can be employed to reduce TMAO production. Small molecules include phenolic compounds such as those extracted from wines (resveratrol) or oranges (feruloylputrescine). Existing cardiorenal medications include but are not limited to aspirin, metformin, and statins, as described above. The TMA lyase inhibitors, betaine reductase inhibitors, and carnitine monooxygenase inhibitors all fall into the category “microbial enzyme inhibition”.

Finally, the plant sterol β-sitosterol has been evaluated for its role in improving atherosclerosis, revealing that it restores several markers related to inflammation, cholesterol, and TMAO production in choline-induced atherosclerosis. Importantly, TMA, TMAO, and FMO3 were all decreased with β-sitosterol treatment, indicating that its effects may not be solely due to TMA lyase inhibition. Treatment also increased gut microbiota abundance and diversity in atherosclerotic mouse models, which was attributed to a reduction in intestinal TMA production.^137^ With these potent effects and a different mechanism than the other TMA lyase inhibitors in development, it is likely that β-sitosterol will continue to be studied for its health benefits. All these novel targets for reducing TMAO have shown that intestinal-produced metabolites hold much more significance in overall health than previously understood. The gut‒heart axis remains of substantial interest, and research continues to be conducted to better understand these connections and how they can be utilized to improve patient outcomes. A summary of the various mechanisms for decreasing TMAO production is presented in Figure 3.

## Discussion

### Paradigm shift in medicine: interconnected metabolomics

The onset of studies into TMAO and its widespread effects on the human body marks a paradigm shift in medicine, with growing interest in gut-derived metabolites such as TMAO reflecting a broader shift toward viewing disease as a product of interconnected metabolic networks. As research is continuing to process pathology in this manner, it is possible to learn from different organ systems and maximize the benefits of research, potentially utilizing treatments with multifaceted benefits, such as those seen in medications that reduce TMAO.

### Controversial role of TMAO

There is ongoing debate in the scientific community regarding both the mechanisms by which TMAO affects the body and its utility as a biological marker in disease. Although the number of studies on TMAO has increased rapidly, its precise role in pathophysiology remains a subject of contention. Some researchers argue that the consistency of experimental data across models, along with the identification of specific molecular pathways, suggests that TMAO plays a causative role in disease progression, making it both a mechanistic insight and a therapeutic target. Critics contend that TMAO's broad associations with inflammation, thrombosis, atherogenesis, and renal function are overstated, proposing instead that elevated levels may simply reflect impaired renal clearance or other underlying disease states—essentially acting as an expensive replacement for creatinine or eGFR testing. Still, neither view is definitive, and the complex interplay between TMAO, cardiovascular health, and renal function makes it difficult to separate causes from correlations without more rigorous, mechanistic, and longitudinal studies. TMAO may function simultaneously as a biomarker and a biologically active compound, with its elevation reflecting dysfunction while still exerting downstream effects. These overlapping roles make the interpretation of TMAO highly context-dependent, underscoring the need for further research to fully understand its clinical value. Until these uncertainties are clarified, the ability to improve patient outcomes through TMAO modulation remains limited.

### Challenges in interpretation

Accompanying the controversy surrounding the role of TMAO, a significant barrier within the current research lies in the host, as no two individuals in the studies have identical microbiota, making it difficult to draw conclusions from the studies. There are also differences in host enzyme makeup, genetic predisposition to drug response, and dietary preferences. This makes human studies challenging, especially when attempting to use dietary intervention as an independent variable or treatment. Additional randomized controlled clinical trials are needed to address the current uncertainty in interpreting and disseminating study results.

Another barrier in the current landscape of the field is the potential for off-target effects using the mechanisms described here. For example, some of the described treatments alter the prevalence and diversity of the microbiota, as well as enzyme regulation. Given the numerous known effects, it is likely that some unknown effects are also at play, and these may not be fully understood until further research is conducted. It is assumed that some treatments target the microbes within the intestine but have no effect on the host, but this may not be true. Only longitudinal, randomized clinical trials in humans can provide the certainty needed to implement these robust treatments in clinical settings.

An important limitation is that many existing studies rely on cross-sectional fasting plasma TMAO levels. As discussed, TMAO elevations frequently display delayed postprandial kinetics, with peak levels often occurring 12–48 h after substrate ingestion. Therefore, single fasting measurements may not fully reflect an individual's average serum levels or capacity for TMAO generation. Ideally, standardized dietary challenge protocols with postprandial assessments would provide a more robust measure. In the absence of such data, findings should be interpreted with caution.

In clinical practice, a final challenge lies in determining whether TMAO provides prognostic value beyond existing markers and whether its measurement has a meaningful impact on clinical management. As TMAO has only recently been available for diagnostic and prognostic sampling, currently available data may not be sufficient for in-depth analysis. Additionally, more questions arise about how this problem might be addressed, as discussions of dietary interventions have consistently been met with skepticism, likely owing to the issues encountered in the modern management of metabolic dysfunction in the United States. Essentially, a problem of knowing the solution but not having a viable way to ensure that the dietary countermeasures are taken correctly may be on the horizon.

Nevertheless, the growing interest in gut-derived metabolites reflects a shift toward a different type of thinking in medicine, where diseases are viewed as a complex interplay of metabolic signals, and each organ plays a role in both the pathophysiology and the potential treatment regimens available. TMAO provides a fascinating example of a “cornerstone” structure, with evidence demonstrating its widespread effects on health in numerous patients. While its role is not yet fully understood, it continues to offer a compelling lens through which to view cardiometabolic, renal, and intestinal pathophysiology as well as the various medications that may influence them (Table 1).

**Table 1.** Classes of treatments to reduce TMAO production.

Drug class	Mechanism	Examples	Efficacy
TMA lyase inhibitors (CutC/D)	Inhibit TMA production from choline	DMB, IMC, and FMC	DMB reduced TMAO levels by about 60% in mice on “Western diet.”^99^
Carnitine reductase inhibitor	Inhibit TMA production from carnitine	Meldonium, quercetin, and feruloylputrescine	After 2 weeks carnitine pre-treatment, Meldonium administration along with carnitine did show decreased TMAO at 1, 2, 4 h.^118^
Microbiome disrupters	Decrease microbes available to make TMA	Antibiotics, metformin, statins, and β-sitosterol	>90% decrease in TMAO induced by phosphatidylcholine challenge after broad-spectrum antibiotic use.^138^ ^,^ ^139^
Renal protective agents	Improve long-term renal function to filter TMAO	ACE Inhibitor/ARB	Two weeks on enalapril reduced rat serum TMAO by about 40%, while increasing TMAO urinary excretion.^89^
Transport modulators	Change renal excretion rate	Meldonium	Meldonium can decrease serum TMAO levels and promote cardioprotective effects via decrease carnitine reabsorption^118^ Other transport modulators have not shown this benefit.^140^
FMO3 inhibitors	Inhibit TMAO production from TMA	Indole-3-carbinol	I3C (consumption of 300 g of Brussel sprouts every day) decreased TMAO between 15 and 20%.^125^
Lifestyle approaches	Alter microbiome or decrease precursors	Weight loss, exercise, vegan or vegetarian diet, and fasting	One week on a vegan diet decreased mean TMAO by about 50%.^64^
Surgical methods	Change the intestinal anatomy	Gastric bypass, Roux-en-Y	Meta-analysis revealed significant increase in TMAO following bariatric surgeries.^141^
Prebiotics/probiotics	Introduce beneficial microbes or fibers	*Lactobacillus, Bifidobacterium*	*Lactobacillus* and *Bifidobacterium* subspecies decreased high-choline diet serum TMAO by about 20%.^129^
Fecal microbiota transplantation	Introduce new microbiota from healthy individuals to change the overall microbiome.	FMT capsule	Fecal microbiota transplantation from lean vegans to patients with metabolic syndrome did not yield significant change in gut TMAO production.^127^
Phenolics/flavonoids/sterols	Alter microbiome or host enzymes	Berberine, resveratrol, quercetin, and β-sitosterol	Resveratrol decreases intestinal production of TMA after choline supplementation by about 60%.^113^

## Future directions

### Data utilization

Given that existing data points have not been thoroughly analyzed, it is imperative to maximize the information that can be gained from them. Some fundamental aspects of the current utility and ordering habits of clinical TMAO testing can provide insight into the prognostic value that TMAO may offer at this point. Tracking trends based on that information can reveal how the value has changed over recent years and how the medical community is adapting to recognize the broader significance of TMAO. The specialties and subspecialties that order it may reflect which fields it has become most relevant to and the diagnoses cited or other labs drawn concurrently may help shed light on the meaning behind its ordering and clinical utility. Along with analyzing the existing data, longitudinal measurements can lead to a better understanding of real-world TMAO measurements and how they trend in patients.

### Longitudinal monitoring

Given the significant cardiovascular and renal associations, it would be interesting to follow patients with serial TMAO measurements and serial echocardiography or renal function measurements to evaluate their correlation in real-world patients. It would also be valuable to examine more targeted assessments, such as CT calcium scores or coronary catheterizations, given that disrupted calcium and cholesterol dysregulation are known effects of elevated TMAO. The longitudinal relationship between TMAO and other data may help clarify correlations in real-world settings, especially for in-depth analysis of different treatments and their effects on TMAO.

### Medication effects

Using existing real-world data, it would be important to analyze this correlation with medication regimens. There is a need to study how existing medications influence TMAO excretion and investigate the links between TMAO levels and GLP-1 receptor agonists, particularly in the prevention of patients without overt disease. Additionally, recent studies have provided some insights into the channels through which TMAO is excreted; however, further evaluation and mechanistic refinement is required. Existing medications may influence this excretion, so it may be essential to conduct retrospective studies on patients with TMAO laboratory values and exposure to medications of interest.

### Novel therapies

In addition to current medications, a more mechanistic analysis of novel therapies would help us understand the molecules, their effects, and how to best mitigate them to improve patient outcomes. For TMA lyase inhibitors, capturing high-resolution bound inhibitor–enzyme structures for the known TMA-producing microbes will help us better understand the mechanisms by which they act. Utilizing other high-throughput virtual screening methods can also discover structures that may have an even greater effect on inhibiting these enzymes. It would be beneficial to have all the bacterial TMA-producing enzyme structures defined, enabling improved screening methods that could yield better overall enzyme inhibition and thus a significant breakthrough in the efficacy of the selected molecules.

These novel therapies can also be tested in scenarios beyond those currently documented in the literature. As discussed, some TMA lyase inhibitors have even shown efficacy in reducing the rate of neurodegeneration in transgenic mice. The only way to determine their limits is to increase the research and explore more options. Examples of fields with untapped potential are cancer, immune dysfunction, and other neurodegenerative diseases.

### Need for clinical trials

Perhaps the most important next step for novel TMAO therapies is randomized controlled trials. These are essential for evaluating how TMAO modulation affects clinical outcomes, particularly in conjunction with medications already in use. This is crucial in translating research from the bench to the bedside, providing patients with the opportunity to benefit from the research. Implementing postprandial measurements for precursor-induced TMAO challenge may provide a more accurate assessment of TMAO production potential and actual patient serum load. This would also increase our understanding of the current controversial aspects of TMAO and provide more information to inform future research.

## Conclusion

In conclusion, TMAO is a simple molecule with a complex relationship with human health. It has various routes of ingestion but limited routes of metabolism and excretion. There have been significant associations made between it and different disease states, including cardiovascular, renal, gastrointestinal, neurological, immunological, and other health areas, which suggests that we stand to benefit significantly from further studying it. As it stands, the evidence suggests that TMAO is a metabolically active chemical with significant effects, which should be further elucidated and targeted for potential therapeutic applications in the future.

## Data Availability

No primary research data.

## Data deposition

No primary research data.
